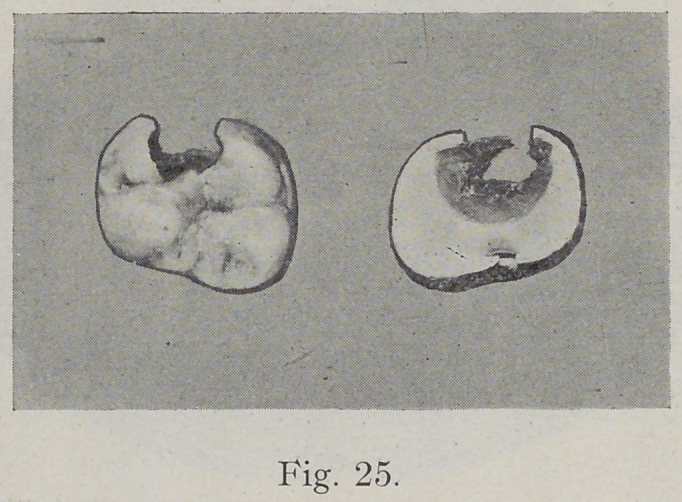# The Preparation of Enamel Walls and Margins for Fillings

**Published:** 1904-09-15

**Authors:** L. P. Bethel

**Affiliations:** Columbus, Ohio


THE
DENTAL REGISTER.

Vol. LVIII. September 15, 1904. No. 9.

### THE PREPARATION OF ENAMEL WALLS AND MARGINS FOR FILLINGS.

BY L. P. BETHEL, M.D., D.D.S., COLUMBUS, OHIO.

Read before the Ohio State Dental Society, December, 1903, and printed from advance proofs by courtesy of the "Dental Summary."

Dr. G. V. Black first called attention to the cleavage of enamel in 1891, but comparatively few followed his teachings, and not until within the past few years has the subject received the recognition it deserves. Dr. F. B. Noyes, supplementing the work of Dr. Black, has presented the various phases of enamel cleavage in several society meetings, but I believe the subject has never been presented before this body.

To make a filling as nearly perfect as possible, the cavity walls should be as strong as can be obtained, and to obtain the strongest walls one should at least have a knowledge of the general direction of the enamel rods in the various portions of the teeth, an understanding of carious action, and possess good judgment as to how much tooth substance should be removed.

In order to present the subject in a systematic and progressive manner, I will divide it into sub-topics as follows:

*First.* Structure of enamel.

*Second.* Carious action on enamel substance.

*Third.* Cleavage of enamel.

*Fourth.* Obtaining of greatest strength in enamel walls and margins.

STRUCTURE OF ENAMEL.

Enamel, as you know, is made up of two calcified structural elements, the enamel rods or prisms, and the interprismatic or cementing substance.

The prisms are long, slender, five or six-sided prismatic rods, built up in layers, alternately constricted and expanded, as will be seen in Fig. 1.

Fig. 1.

For all the constrictions and expansions of the enamel seem to more often come opposite each other than to interlock, the spaces are filled with the prismatic cementing substance and the whole held in close union. The cementing substance, however, is not as strong as the enamel rods themselves, and is more readily acted upon by acids than the prisms, as will be shown later.

A cross-section of enamel, as seen in Fig. 2, has somewhat the appearance of a mosaic floor.

For our purpose it is not necessary to go into the histological structure of the enamel in detail, so with this

Fig. 2.

superficial view of it we will pass to the next division of the subject, leaving the structural arrangement of the rods, etc., to be considered later.

### CARIOUS ACTION ON ENAMEL SUBSTANCE.

Caries attacks the surface of enamel and is looked upon

Fig. 3.

by Miller and others as a chemico-parasitic process. Williams has found bacterial masses closely adherent to the enamel surface of the tooth and demonstrated that caries occurs directly beneath these gelatinous placques. Fig. 3

Fig. 4.

is a reproduction of a photo-micrograph showing a mass of bacteria attached to Nasmyth's membrane on the proximal side of a bicuspid tooth.

Fig. 5.

Bacteria may, or may not, cause caries. This depends upon conditions of nutrient media in the mouth. In the presence of proteids and absence of carbohydrates, the resulting putrefactive process would bring about an alkaline reaction which would not affect the enamel; but in the presence of carbohydrates (sugars, starches, etc.,) fermentation takes place, resulting in the formation of an acid by the bacteria, supposed to be lactic acid, that does act on the tooth enamel, preparing the way for caries.

Carbohydrates being constantly present—the mouth, and carbohydrate fermentation taking precedence over proteid and other kinds of fermentation—you will readily

Fig. 6.

see the great liability of the teeth to carious action.

As stated, the interprismatic cementing substance is weaker than the enamel rods themselves and is more readily acted upon by the acid excreted by the bacteria adherent to the enamel surface. Fig. 4 shows a highly-magnified mass of bacteria attached to the surface of a tooth and the action of acid in dissolving the cement substance and forming V-shaped spaces between the enamel rods. Fig. 5

Fig. 7.

shows the appearance of the surface of enamel under one of these bacterial placques. The acid has attacked the interprismatic substance and in some places the ends of the rods have broken away. Fig. 6, a section of enamel showing deeper penetration of the excreted acid between the enamel rods. Fig. 7 shows further progress of caries

Fig. 8.

and deeper penetration into the enamel substance. Fig. 8 is a section, where the bacterial mass has been carefully removed, showing the action of the acid in general on the enamel. Williams says: "As the acid produced by the micro-organisms dissolves out the cement substance between the rods, the bacteria insinuate themselves deeper and deeper in the depressions thus formed. The acid is continually forming, and its full dissolving power continuously acting on the tissue. With the removal of the cement substance the loosened rods and sections fall apart, and the work of destruction is finished." Fig. 9 shows the final disruption and destruction of enamel rods, the result of the dissolution of the cementing substance by the acid.

Fig. 9.

CLEAVAGE OF ENAMEL.

If you have not already noticed you will see upon close observation that enamel usually checks or cracks in definite directions. The ground specimen, Fig. 10, shows a number of checks. You will observe that these cracks are all in the same direction and in the direction of the enamel rods in general.

The interprismatic cementing substance is weaker than the enamel rods and enamel will usually break at the cementing substance before it will break in the rods themselves.

Fig. 10.

For instance, where there is an opening in the enamel substance, made by caries or otherwise, the enamel along the sides of this cavity is readily broken down by a blow or pressure from a sharp chisel held at an acute angle to the surface of enamel, on account of the cementing substance giving way under the wedging force of the chisel. In this way it is easy to break down any extent of enamel, while if one attempts to cut across the rods he finds it a difficult task, owing to the enamel composing the rods being so dense and hard. At times, however, the enamel is not so readily forced apart, and in fact it seems almost impossible to break or cut it away in any manner, and when it does break it is irregular and not in the definite directions shown in Fig. 10.

Searching for a cause of this, we find that the structural arrangement of enamel is not all the same. In some sections the rods run straight from the surface of the enamel to the surface of the dentine. In other sections we find the rods straight a part of this distance and then they take a twisted or wavy course. In other sections we find throughout the whole extent that the enamel rods run irregularly and have this wavy and gnarled appearance. In cleaving enamel, then, we may find enamel with straight rods, enamel with straight and gnarled rods, or enamel gnarled throughout its whole extent. Fig. 10 is a section of straight enamel, easy to cleave in definite directions along the line of the enamel rods. Fig. 11 is a section of gnarled enamel impossible to cleave along the line of the rods, and difficult to cleave in definite directions. It is like splitting a pine knot, and many of the enamel rods are broken across. In fact, at times this gnarled enamel seems to defy the best tempered chisel or bur.

Fig. 11.

The location of gnarled enamel is usually on the occlusal surfaces of bicuspids and molars, and over the cusps of the teeth. The straight enamel is found on the labial, buccal, lingual and in fact, all axial surfaces. At times, we find the outer portion of enamel on the occlusal surface made up of straight rods, while the inner ends of the rods are gnarled. In attempting to cleave this sort of enamel, one usually finds that the enamel cleaves away as far as the straight rods extend, then breaks away, leaving the gnarled portion adhering to the dentine.

In attempting to cleave gnarled enamel, one will find that it does not break in definite directions, but irregularly across the rods as well as between them, and has to be planed down to a smooth edge with a chisel to make a good margin.

The straight enamel cleaves in the direction of the rods and requires but little planing to obtain a smooth margin. It is probably impossible to cleave a portion of enamel without breaking across some of the rods, but cleaving from the surface with chisel, at an acute angle, leaves the portions of rods near the inner ends supported by dentine and enamel and protected by the subsequent filling.

The object of cleavage of enamel is to ascertain the direction of the rods in the location of cavity margins, that these margins may be supported by full length or well-supported enamel rods, so far as possible.

Dr. Black has given the following requirements for greatest strength in cavity walls:

1. Enamel should be supported on sound dentine.

2. Rods which form the cavo-surface angle should run uninterrupted to the dentine and be supported by short rods with their inner ends resting on the dentine and their outer ends abutting the cavity wall where they will be covered by the filling.

3. The cavo-surface angle should be cut in such a way as not to expose the ends of the rods to fracture in condensing the filling against them.

To cleave enamel successfully, the chisel should be sharp and the enamel broken down little by little. If the chisel has not a "biting" edge, but is dull, many rods are broken across and the resulting break does not follow so nearly the direction of the enamel rods.

Fig. 12 shows a section of straight enamel as cleaved by a sharp chisel. It will be seen that the enamel has separated in the general direction of the enamel rods and requires but little planing with a chisel to make a good margin.

Fig. 13 represents the margin, as planed with a chisel,

Fig. 12.

following the directions of the enamel rods, and beveled at the cavo-surface angle to protect the ends of the rods from fracture when filling.

Fig. 13.

Fig. 14 is a drawing representing a bucco-lingual section of an upper bicuspid and shows the general direction of the enamel rods in this particular location. It will be noticed that the enamel rods in the gingival portion do not run horizontally from dentine to surface of enamel, as many suppose, but that they incline somewhat gingivally. As we pass from the gingival to the occlusal we see that the rods become horizontal to the surface of the tooth, then gradually incline more and more toward the cusps until at some point on the cusps the rods stand vertically. Passing over the cusps the rods may be seen inclining in the opposite direction toward the dentine, following the curved surface of the cusps, but varying according to the height of the cusps; the higher the cusp, the greater the inclination.

In the cleavage of enamel for strength in cavity walls, one should acquire a knowledge of the general direction of these rods in every location on the tooth, and prove the direction by cleaving with the chisel.

Fig. 15 shows some diagramatic sections of a bicuspid and a central incisor. Fig. 15, A, represents a cross-section

Fig. 14.

of a bicuspid. In the formation of an approximal cavity in such a tooth, you will notice the difference in the angle of cleavage, according to location and extent of the cavity, that should be followed in order to obtain the strongest walls. Fig. 15, B, C, D, E, show the general arrangement of the enamel rods in various locations on an incisor, and the angle of cleavage in the variously located cavities. (This slide was made from one of Dr. G. V. Black's diagrams.)

Now let us look at the application of this cleavage in preparing a few cavities and see if its observance is advantageous.

Fig. 16 shows a cavity prepared by the use of burs as many dentists would shape a cavity of this sort if they were paying no attention to the direction of the enamel rods. After that cavity was prepared, a sharp chisel was applied to the tooth and the enamel cleaved away in the

Fig. 15.

direction shown in Fig. 17. When burs are used in the shaping of cavities, the operator cannot tell anything about the direction of the enamel rods.

Now some will ask, why a cavity prepared like Fig. 16

is not just as strong as one cut away toward the incisal edge as shown in Fig. 17? It is because the enamel rods in this location take the direction indicated in Fig. 17, and shown in Figs. 14 and 15, and if the cavity be cut as indicated in Fig. 16, the outer portion of the lower wall will be made up of short rods, the long rods having been cut across in burring, and these short rods will be unsupported by the dentine and consequently make a weak wall that is liable to fracture or break away in either the filling operation or subsequently, as most operators have observed in cases of this kind.

Fig. 16.

Fig. 17.

Fig. 18.

Fig. 18, the same tooth, shows in pencil the general direction of the enamel rods and how the short rods would be left unsupported. In the gingival third of the tooth the rods incline slightly toward the gum; in the middle third they are more nearly horizontal; in the incisal, or occlusal third they incline incisally, or occlusally, and vary in abruptness of inclination according to the length and amount of curve of the margin of the tooth toward the incisal or occlusal edge.

It must be borne in mind that developmental grooves are also areas of weakness in enamel. If a cavity wall comes almost to such a groove, it is better to extend such cavity to that groove or just beyond to obtain the strongest wall. Fig. 17 might furnish an example of this, for in that specimen the groove is abnormally well marked. If this precaution is not taken, especially where the grooves are well marked, and sometimes along the line of one or both of the grooves on a central incisor, by close inspection one can find a minute check even though the grooves themselves are not prominent, the weakened corner of enamel is apt to be checked or broken away with results seen in Fig. 19.

Fig. 19.

In the preparation of cavities for filling, sometimes it is found that the enamel rods run toward the cavity, and in other instances away from the cavity. On occlusal surfaces the rods incline toward the cavity. This is shown in Fig. 14. After making parallel walls to a cavity in this location, we will find that the cavity walls have the greatest support possible, as the enamel rods forming the cavo-surface angle run uninterrupted to the dentine and are supported by shorter rods with their inner ends resting on sound dentine and their outer ends abutting the cavity wall. Parallel walls in such a cavity are all that is necessary so far as strength is concerned, but to protect the ends of the rods from fracture while making the filling, the cavity margins should be slightly beveled.

Proximal cavities and cavities on other axial walls, however, must be dealt with differently, for the rods incline away from the cavity. On all proximal surfaces we find this inclination of the rods, and if the cavity approaches near the marginal ridge, we have a weak wall at that point. It would insure a stronger operation to extend the cavity into the occlusal portion, making a compound cavity of it.

On the lingual surface of incisors one must watch closely the direction of the enamel rods, especially near the curve

Fig. 20.

at the basal ridge, as illustrated in Fig. 20. Margins of axial cavities must be more carefully beveled than those of occlusal cavities, for the inclination of the rods is greater, and we find more short rods in this location, which must be covered by the filling for protection. Short or partial enamel rods are found on the axial surfaces of teeth, particularly near the marginal ridges, for the crown surface is much greater in area than the dentine surface, and the enamel rods themselves are as large in diameter at their outer as they are at their inner ends, leaving spaces at the outer ends to be filled with partial or short rods.

In the occlusal portions of teeth the opposite will be found, the short rods next to the dentine, especially where the plates of the cusps join, forming a groove. And it seems probable that faults, so often found in the enamel structure at these points, are due to the arrested or incomplete development of the enamel rods owing to the manner of deposit and the peculiar structural arrangement of the surrounding rods.

In cleaving enamel it will be noticed that the enamel is not of uniform thickness on all teeth, but that it varies materially. On some teeth it is thick and on others comparatively thin. On some teeth at the gingival line it appears to be as "thin as paper" we might say for illustration. Fig. 21 shows a few teeth on which some portion of

Fig. 21.

the enamel was cleaved with a chisel. In the incisors, C, D, E, some variation is shown. The enamel at the incisal edge of E is thicker than in like position on C. The enamel over the basal ridge in E and in D is thicker than in similar location in C. On the lingual surface of D, the enamel is heavier throughout than on C. Notice the difference in the thickness of enamel in the bicuspids B and G.

The cavity in A shows the direction of cleavage of enamel in this location. Notice what a great inclination the rods have near the occlusal. A weak wall would be left here if, in the preparation of this cavity, it were not extended over into the occlusal surface. H shows the direction of the rods near the gingival line. F shows direction of almost invisible checks that are here penciled over to make them appear in the picture.

To obtain the greatest strength in cavity walls, sound tooth substance is essential, and the operator in opening the cavity of decay should be sure that he cleaves to sound enamel substance.

Sometimes the acid, excreted by the caries producing micro-organisms, will penetrate through the whole thickness of enamel before the rods give way or fall apart. In other instances the penetration of acid may be only to a partial depth, but enough to weaken the enamel. And again,

Fig. 22.

Fig. 23.

when the bacteria gain entrance to the dentinal tubules and through their branchings cause destruction of the tooth substance laterally as well as direct, we may find an acid disintegration of the enamel rods at their inner ends, through backward caries, weakening the enamel in this section of the tooth, although its outer surface may have every indication of perfect structure.

Fig. 24.

It is therefore important, in the preparation of enamel walls for strength, to cleave beyond the line of acid penetration. Dr. Black's first requirement for strength in enamel walls, viz: "Enamel should be supported on sound dentine," should always be borne in mind in the cutting away of affected enamel structure.

Fig. 22 is a drawing that shows the lateral extension of caries. Fig. 23, a tooth ground to expose the cavity of decay, shows the undermining of the enamel by caries. A semi-decalcified portion of enamel extended quite a distance beyond the cavity proper, and will be seen at "a" in the engraving.

Fig. 24 shows a number of ground teeth where there was but comparatively small opening through the enamel, yet caries is extensive in every direction, and overhanging enamel has little support. Fig. 25 shows the cavity as it appeared in the occlusal surface of a molar and as it was found after the roots had been excised and the tooth ground upward from the roots until the decayed portion was fully exposed, showing the comparative lateral extension of carious action.

Fig. 25.

There is one point more that should not be overlooked in the preparation of cavities for fillings, and that is the beveling of cavity margins.

Cavities need a greater bevel when located on axial surfaces than when on the occlusal, for here one finds more of the short enamel rods.

Using the chisel to plane and shape enamel walls and margins requires great care if a perfect margin be desired. In planing it will be observed that where the enamel rods are more or less broken apart, the margin will have a whitish appearance, for the rods dislodged by the chisel appear as a fine powder. Continue the shaving or planing until the enamel appears clear and vitreous and you will have made as good a surface to the wall as is possible.

If burs are used to shape enamel walls, or bevel the margins, the operator cannot tell anything about the direction of the rods, or whether he is leaving short rods at the outer margin to weaken it.

Do notb evel your cavity margins too extensively or you will find that the finished filling will have a feather-edge along the beveled margin that is liable to be easily curled or raised from the margin. This is apt to occur when disks or strips are used for beveling instead of a sharp chisel.

## Figures and Tables

**Fig. 1. f1:**
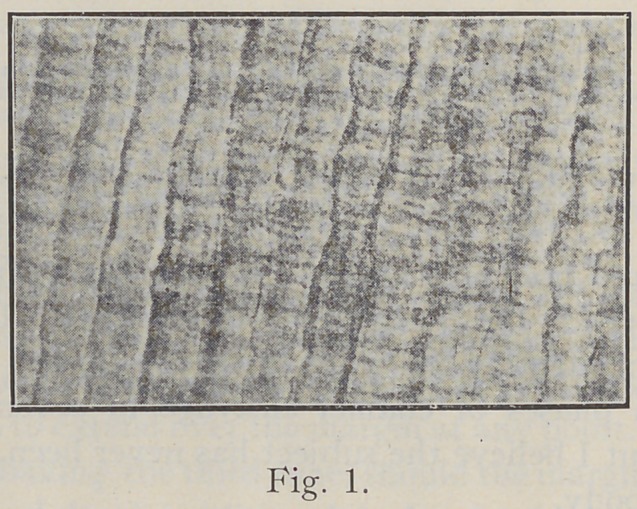


**Fig. 2. f2:**
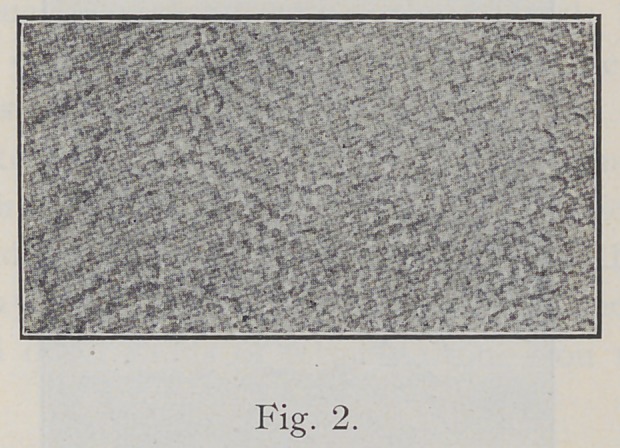


**Fig. 3. f3:**
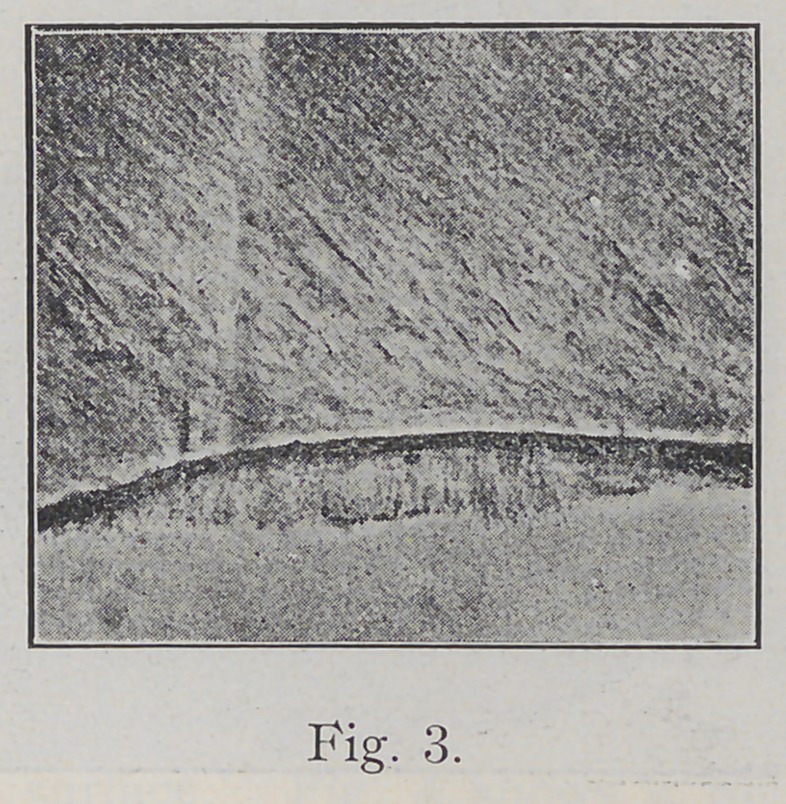


**Fig. 4. f4:**
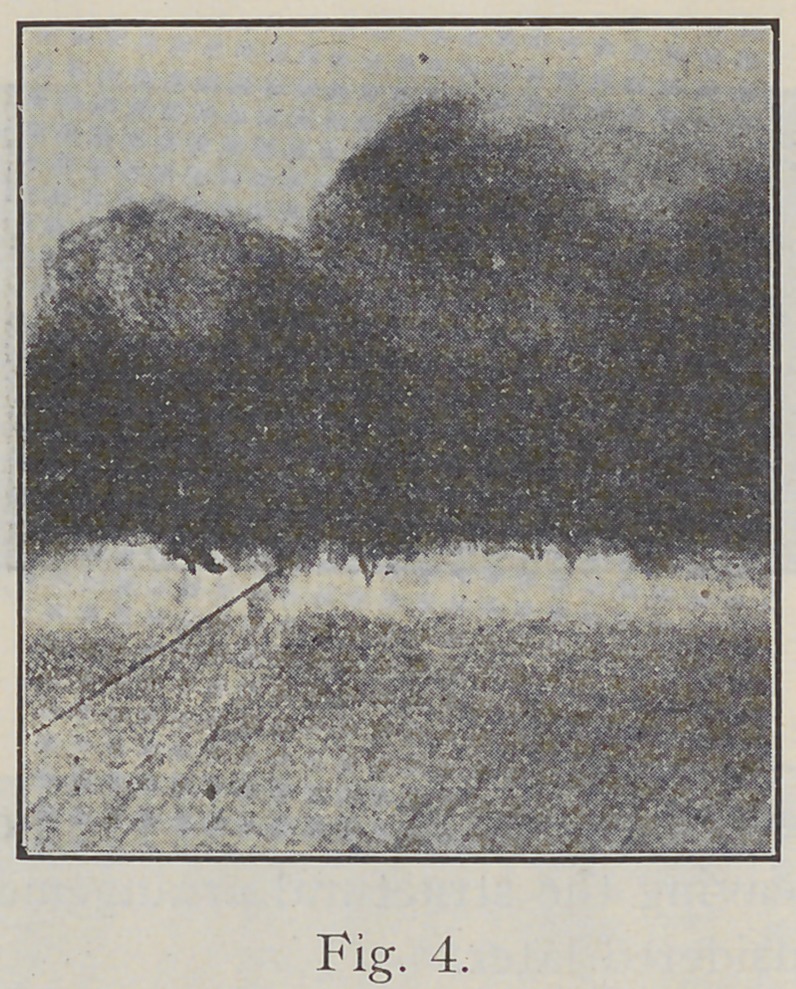


**Fig. 5. f5:**
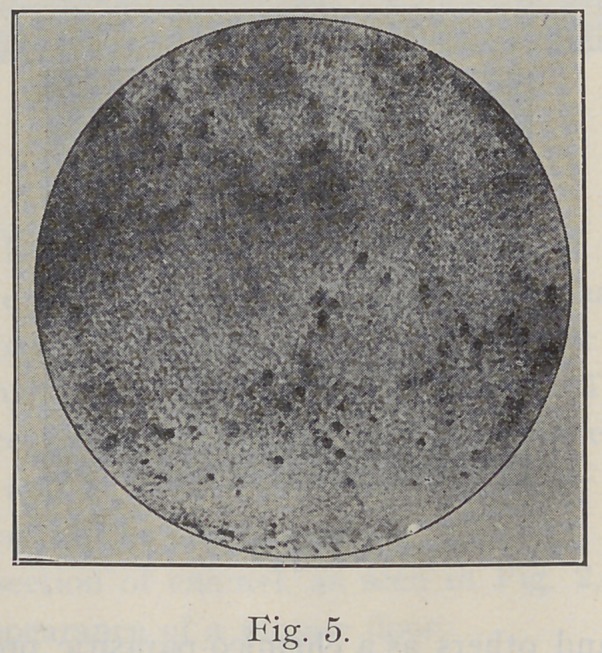


**Fig. 6. f6:**
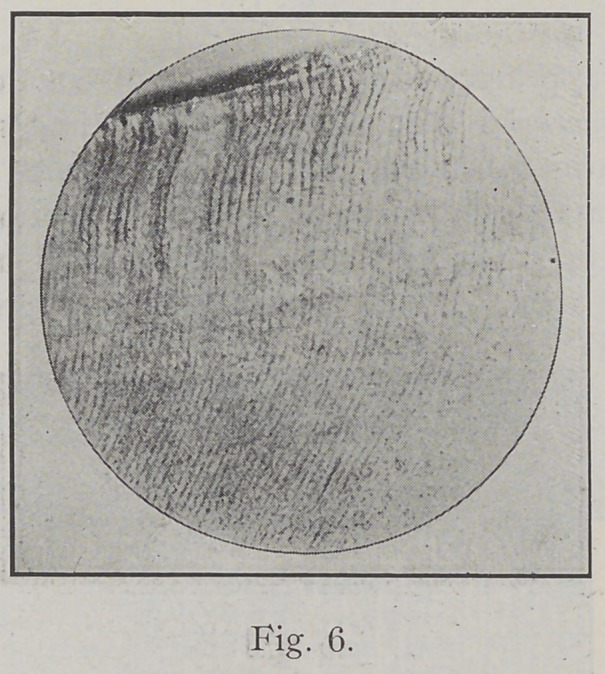


**Fig. 7. f7:**
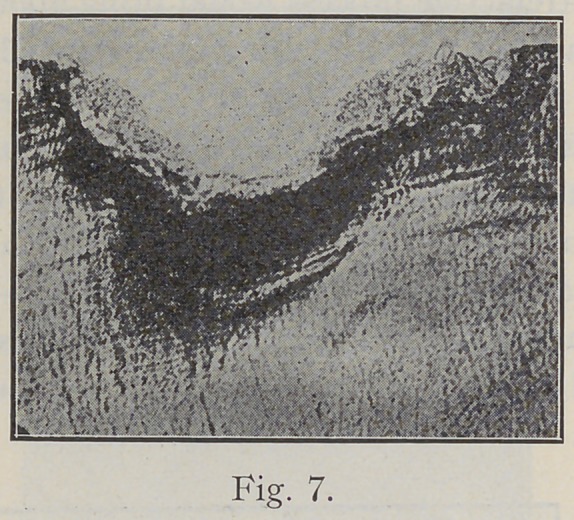


**Fig. 8. f8:**
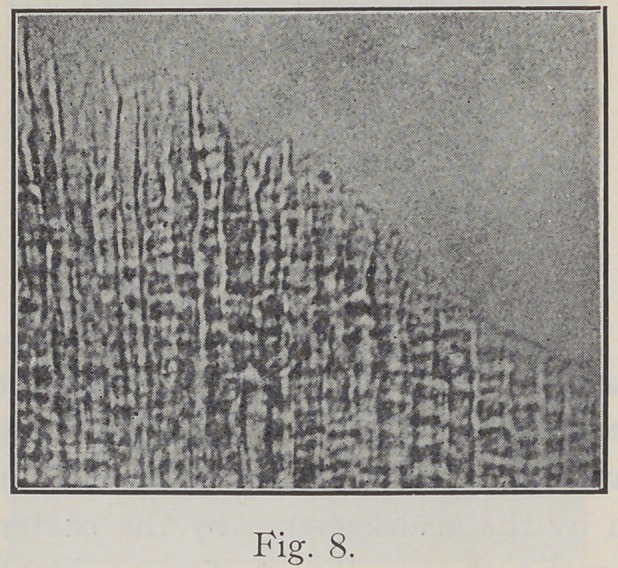


**Fig. 9. f9:**
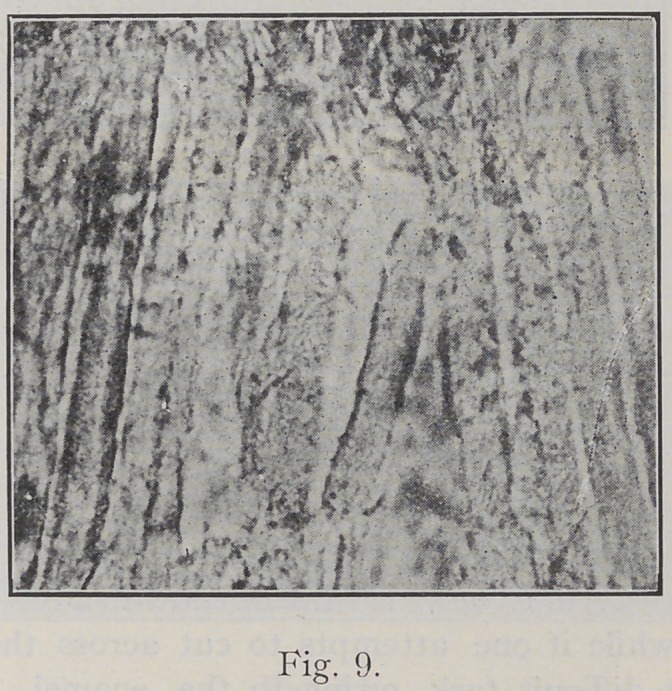


**Fig. 10. f10:**
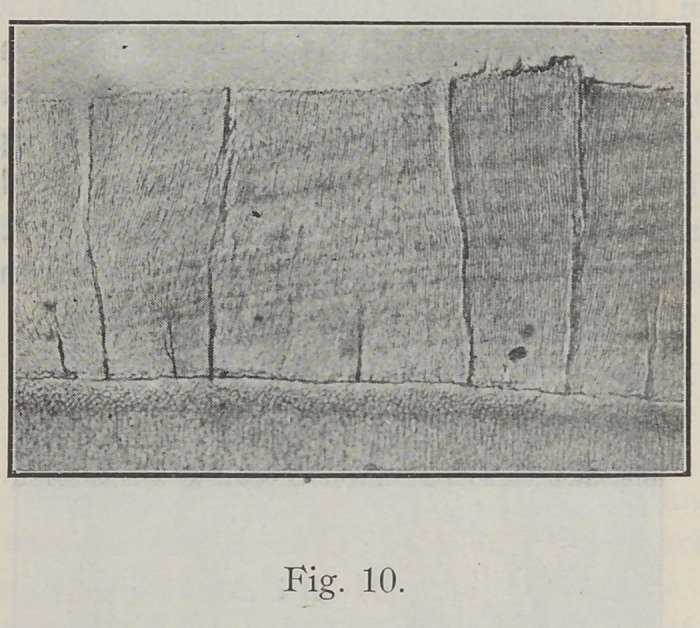


**Fig. 11. f11:**
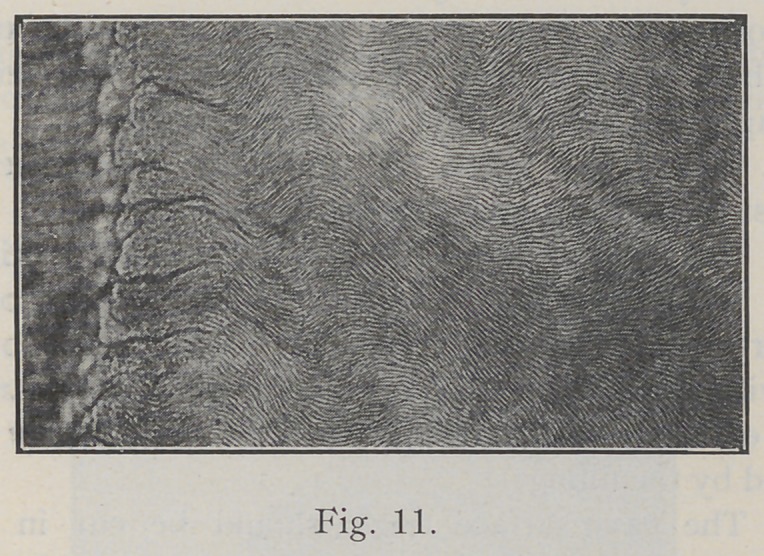


**Fig. 12. f12:**
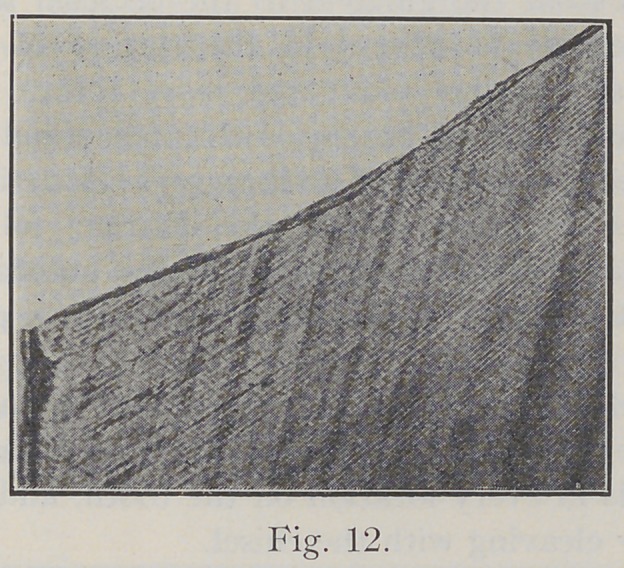


**Fig. 13. f13:**
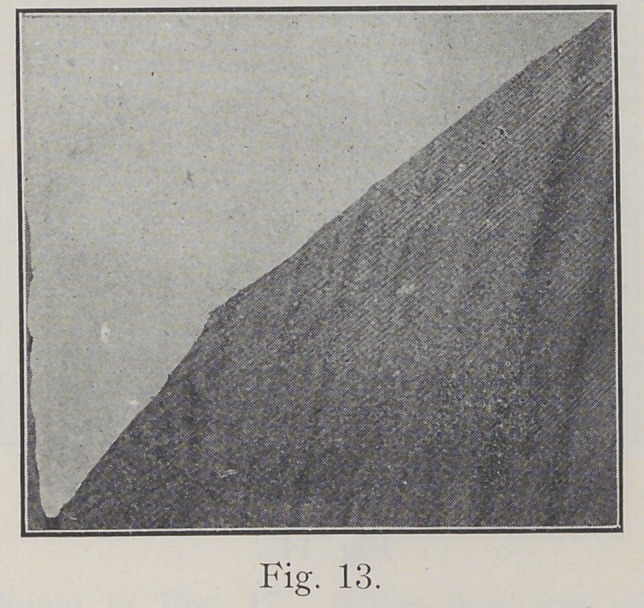


**Fig. 14. f14:**
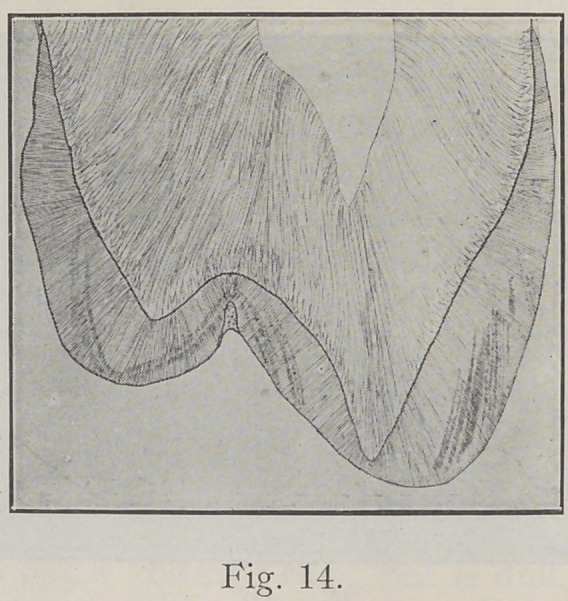


**Fig. 15. f15:**
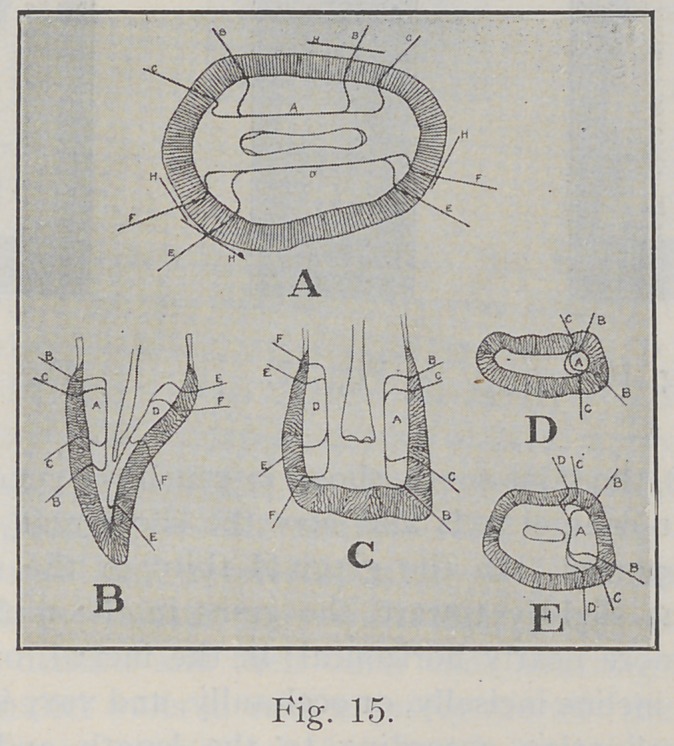


**Fig. 16. f16:**
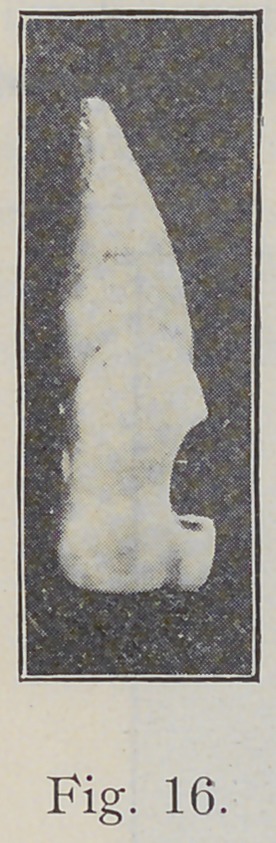


**Fig. 17. f17:**
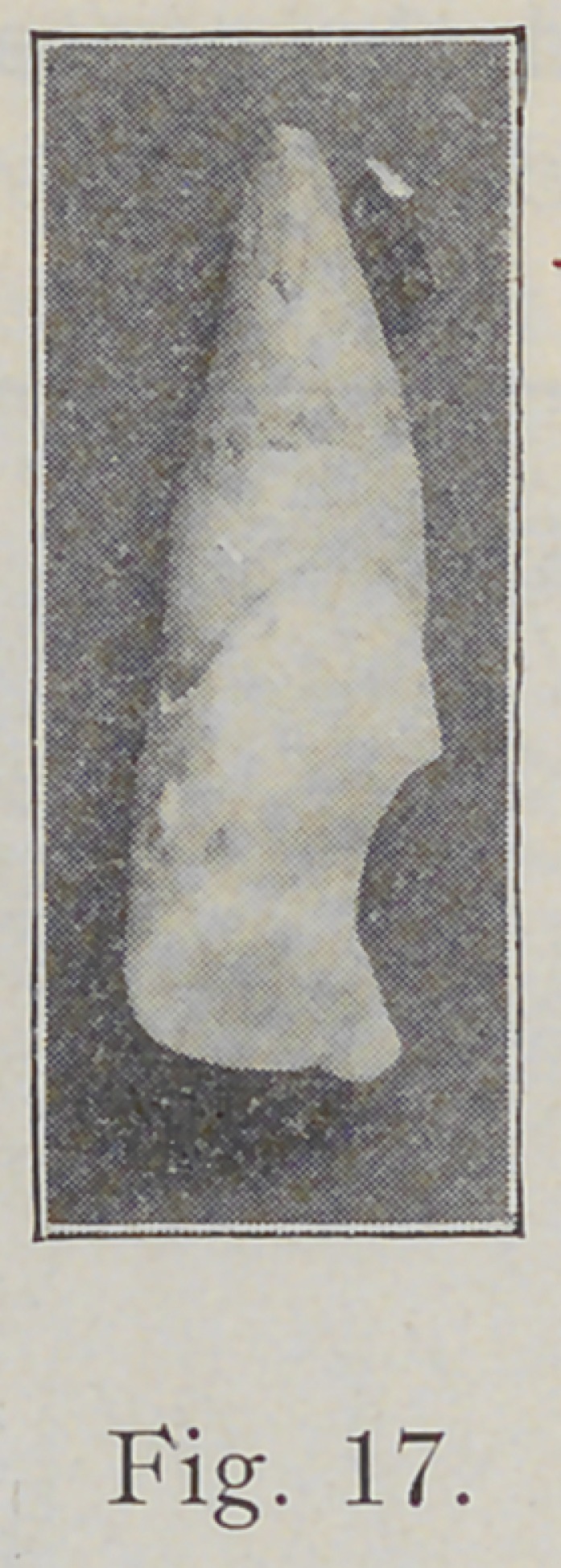


**Fig. 18. f18:**
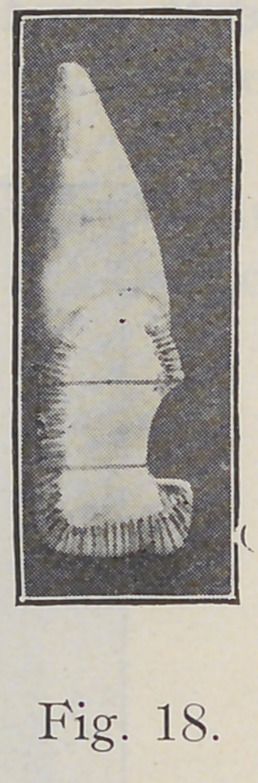


**Fig. 19. f19:**
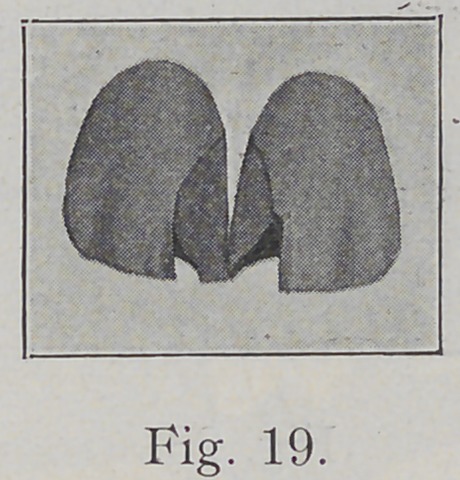


**Fig. 20. f20:**
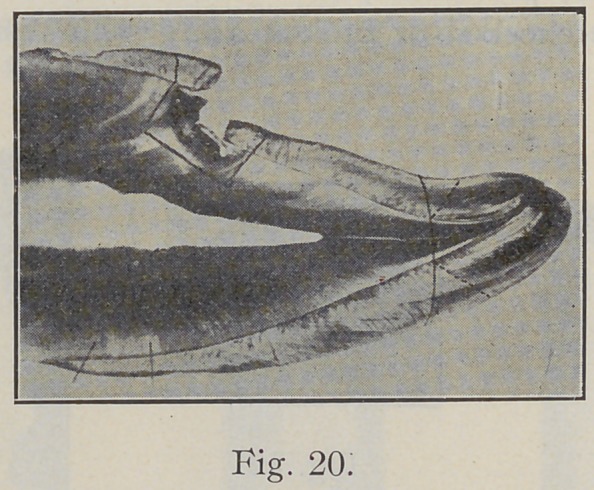


**Fig. 21. f21:**
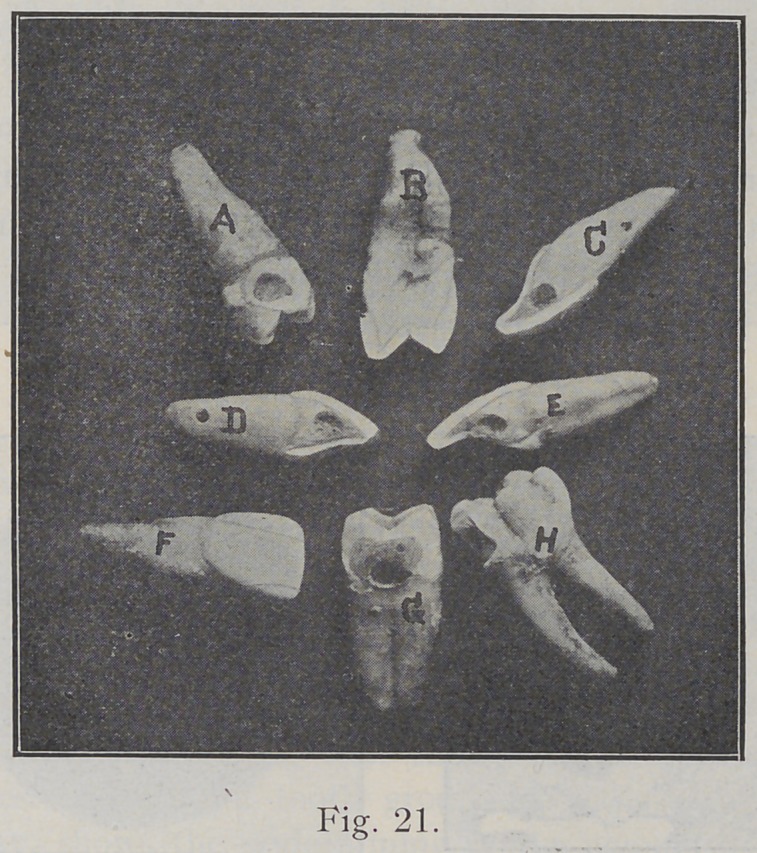


**Fig. 22. f22:**
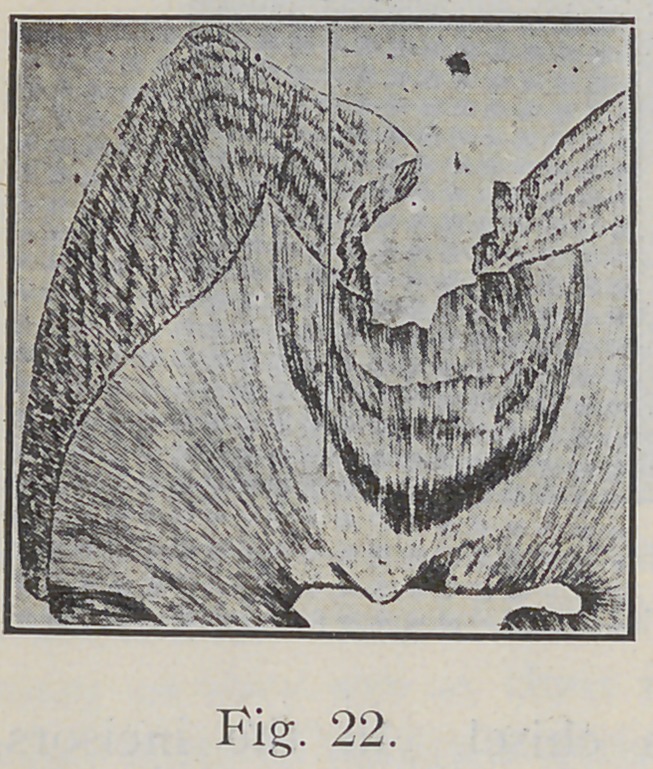


**Fig. 23. f23:**
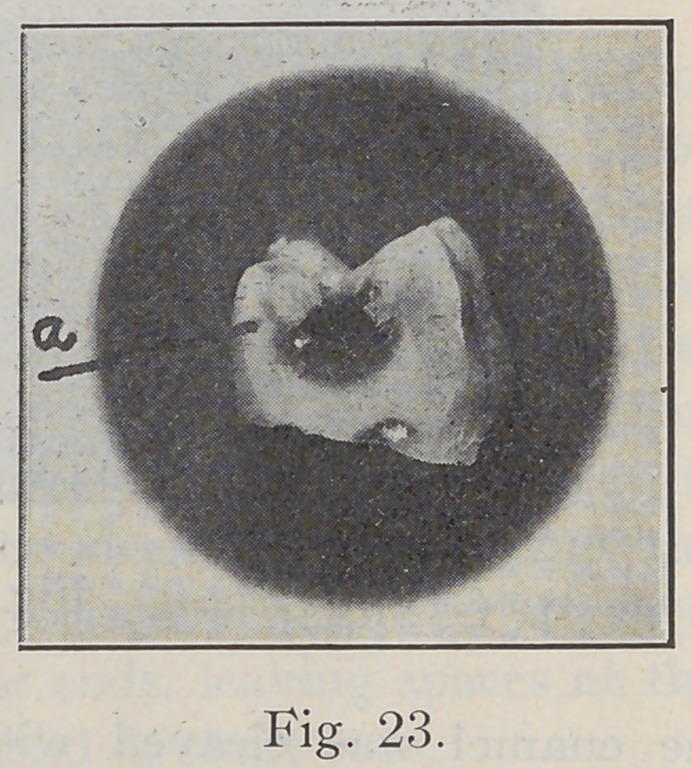


**Fig. 24. f24:**
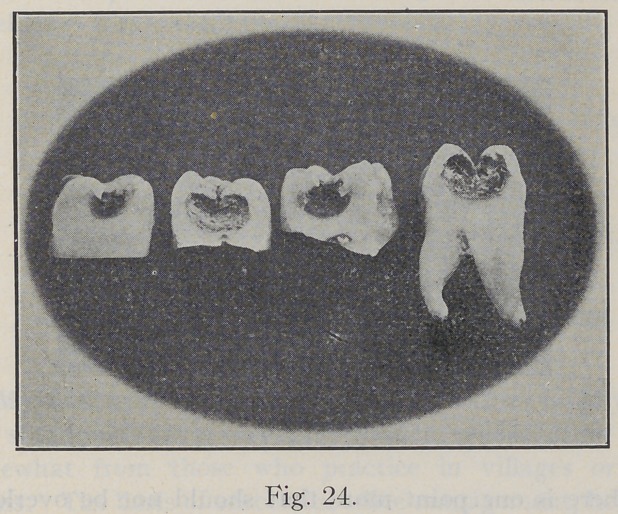


**Fig. 25. f25:**